# BET inhibitors (BETi) influence oxidative phosphorylation metabolism by affecting mitochondrial dynamics leading to alterations in apoptotic pathways in triple‐negative breast cancer (TNBC) cells

**DOI:** 10.1111/cpr.13730

**Published:** 2024-09-02

**Authors:** Teresa Rossi, Egidio Iorio, Mattea Chirico, Maria Elena Pisanu, Nicola Amodio, Maria Eugenia Gallo Cantafio, Ida Perrotta, Francesca Colciaghi, Marco Fiorillo, Alessia Gianferrari, Noemi Puccio, Antonino Neri, Alessia Ciarrocchi, Mariaelena Pistoni

**Affiliations:** ^1^ Laboratory of Translational Research AUSL‐IRCCS di Reggio Emilia Reggio Emila Italy; ^2^ High Resolution NMR Unit Core Facilities, Istituto Superiore di Sanità Rome Italy; ^3^ Department of Experimental and Clinical Medicine University Magna Graecia of Catanzaro Catanzaro Italy; ^4^ Department of Biology, Ecology and Earth Sciences Centre for Microscopy and Microanalysis (CM2), University of Calabria Cosenza Italy; ^5^ Epilepsy Unit Fondazione IRCCS Istituto Neurologico Carlo Besta Milan Italy; ^6^ Department of Pharmacy, Health and Nutritional Sciences University of Calabria Rende Italy; ^7^ Scientific Directorate AUSL‐IRCCS di Reggio Emilia Reggio Emila Italy

## Abstract

Repressing BET proteins' function using bromodomain inhibitors (BETi) has been shown to elicit antitumor effects by regulating the transcription of genes downstream of BRD4. We previously showed that BETi promoted cell death of triple‐negative breast cancer (TNBC) cells. Here, we proved that BETi induce altered mitochondrial dynamics fitness in TNBC cells falling in cell death. We demonstrated that BETi treatment downregulated the expression of BCL‐2, and proteins involved in mitochondrial fission and increased fused mitochondria. Impaired mitochondrial fission affected oxidative phosphorylation (OXPHOS) inducing the expression of OXPHOS‐related genes, SDHa and ATP5a, and increased cell death. Consistently, the amount of mitochondrial DNA and mitochondrial membrane potential (∆Ψm) increased in BETi‐treated cells compared to control cells. Lastly, BETi in combination with Metformin reduced cell growth. Our results indicate that mitochondrial dynamics and OXPHOS metabolism support breast cancer proliferation and represent novel BETi downstream targets in TNBC cells.

## Graphical abstract

Pharmacological targeting of the transcription factor BRD4 by BET inhibitors (BETi) affects mitochondrial dynamics. Specifically, BETi alter the fusion/fission equilibrium and OXPHOS metabolism by changing the expression of specific targets: DRP1 and SDHa, respectively. These alterations reduce cell proliferation and induce cell death.

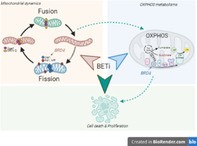

## 1 INTRODUCTION

Breast cancer (BC) is one of the most prevalent malignant tumours in women and the second cause of cancer‐related mortality in females worldwide.^1^ Among those, triple‐negative breast cancer (TNBC) is the most aggressive subtype with limited treatment options^2^ and lacks the expression of oestrogen receptor alpha (ERα), progesterone receptor, and human epidermal growth factor receptor 2. bromodomain‐containing protein 4 (BRD4), a member of the bromodomain and extra‐terminal domain (BET) family, acts as a chromatin reader to regulate transcription by linking acetylated histones and core components of the transcriptional apparatus.^3^ Our group and others have shown that BET inhibitors (BETi), such as JQ1 and OTX015 (thereafter named OTX), suppress the growth of multiple types of tumours both in vitro and in vivo.^4^, ^5^ Induction of apoptosis BETi‐activity‐mediated can be regulated by the mitochondrial pathways.^6^, ^7^, ^8^ Mitochondria are organelles involved in key cellular functions, including energy production and cell death regulation,^9^, ^10^, ^11^, ^12^ which respond to different physiologic or stress stimuli by adapting their structure and function.^13^ Most important structural changes are represented by the mitochondrial fission and fusion phenomena, which occur both in normal and cancer cells. Mitochondrial fission ensures an adequate number of mitochondria to support growing and dividing cells. Besides, it participates in the replacement of damaged mitochondria through mitophagy, therefore representing and serving as a quality control mechanism for the cells.^13^, ^14^, ^15^ Mitochondria fusion, on the other hand, is required to maximize ATP production when cell metabolism needs to rely on oxidative phosphorylation (OXPHOS), or when mitochondria react to stress stimuli. In these cases, mitochondria appear as elongated healthy organelles that complement the dysfunctional mitochondria.^16^, ^17^, ^18^, ^19^ Mitochondrial fusion enhances OXPHOS while mitochondrial fission promotes glycolysis.^20^ Several studies reported that BETi treatment in solid tumour cells perturbs mitochondrial dynamics and function.^21^, ^22^, ^23^ However, it is still to be clarified whether this effect is determined by an overall BETi‐induced metabolic reprogramming or by a direct effect on mitochondria structure and function. Besides, very little is known about the effect of BETi on mitochondria in TNBC cells. Thus, although the alteration of mitochondrial dynamics and metabolism is relevant in the pathophysiology of several diseases^24^, ^25^ and is common in all the BC cells upon BETi administration, the identification of molecular mechanisms that mediate mitochondrial alterations may provide new insights and open the path to novel TNBC therapeutic regimens. Here, we demonstrated that BETi‐treatment enhances fusion and triggers profound metabolic variations, which contribute fundamentally to the proliferation/apoptosis imbalance and might constitute promising novel therapeutic targets.

## 2 MATERIALS AND METHODS

### 2.1 Cells and drug treatments

Human TNBC MDA‐MB231 and Hs578t cells were purchased from the American Type Culture Collection. Human TNBC BT549 was obtained from Dr. Bonetti, IFOM‐IEO Campus, Milan. Human luminal BC MCF7 was purchased from Sigma‐Aldrich. All the TNBC cell lines were cultured in DMEM (Life Technologies) at 37°C/5% CO_2_ in a medium supplemented with 10% fetal bovine serum (FBS, Thermo Fisher Scientific, Waltham, MA, USA) and 1% penicillin–streptomycin (P/S, Euroclone, Milan, Italy). Hs578t medium was supplemented with 0.01 mg/mL human insulin (Merck Sigma‐Aldrich). MCF7 was cultured in EMEM (Life Technologies) at 37°C/5% CO_2_ in a medium added with 10% FBS, 1% P/S, and 1% Non‐essential Aminoacids (NEAA, Life Technologies). All cell lines were routinely tested for mycoplasma and authenticated by SNP profiling at Multiplexion GmbH (Heidelberg, Germany) in 2023. Subconfluent cells were treated with DMSO (referred to as CNT, Merck Sigma‐Aldrich), JQ1 (Merck Sigma‐Aldrich), OTX‐015 (referred to as OTX, Medchem), Metformin (referred to as Met, Merck Sigma‐Aldrich) and Mdivi‐1 (Mitochondrial Division Inhibitor 1, Merck‐Sigma‐Aldrich). Drugs were used at different time points and concentrations depending on the specific assay. Specific information can be found in figure legends.

### 2.2 Drug synergism quantification

Drug combination studies and synergy quantification were realized with CompuSyn software based on the Chou‐Talalay method. Dose‐effect curves were determined by counting viable cells after 96 h of JQ1, OTX (BETi), and Metformin (Met) treatment. At least three different concentrations of each BETi drug (from 0.02 to 1 μM) were combined into three concentrations of Met (from 0.5 to 5 mM).

### 2.3 Cell proliferation

MDA‐MB231, Hs578t, and BT549 cells (2 × 10^3^/well) were seeded in 96‐well plates (Corning). The day after, the cultures were re‐fed with a medium containing either CNT (DMSO or H_2_O) or BETi, Met, or Mdivi‐1. Plates were placed in the IncuCyte S3 Live‐Cell Analysis System (Essen BioScience), equipped with a 10× objective in a CO_2_ incubator at 37°C. Proliferation was measured using real‐time, phase‐contrast images or IncuCyte NucLight Rapid Red Reagent (Sartorius) according to the manufacturer's instructions. For cell counting of BRD4 siRNA, cells were trypsinized, washed, and suspended in phosphate‐buffered saline (PBS, Euroclone) every day. Cell count was performed by a Trypan blue (Sigma‐Aldrich) staining and using the Invitrogen Countess 3 (Thermo‐fisher).

### 2.4 Reactive oxygen species (ROS) measurement

Dihydroethidium (DHE) assay was performed according to the manufacturer's protocol (Abcam, cat# ab236206) following MDA‐MB231 and Hs578t cells treatment for 24 hs with BETi 1 μM, positive and negative (NAC, N‐acetylcysteine, Merck Sigma Aldrich) controls were supplied with DHE assay kit. Fluorescence was measured using the microplate reader GloMax Discover Microplate Reader (Promega) at 520 nm (excitation) and 580–640 nm (emission). The fluorescence was normalized to the total number of cells.

### 2.5 Mitochondria metabolic activity (MTT) and cell death detection

For the measurement of metabolic activity and cell death, the assays were conducted according to the previous study.^4^

### 2.6 Transfection of siRNA and plasmids

For siRNA transfection: MDA‐MB231, BT549, and Hs578t cells were reverse‐transfected with RNAiMax Lipofectamine (Thermo Scientific, Waltham, MA, USA) according to the manufacturer's instructions. siRNA validation was collected 24–48–72–96 h after transfection. siRNAs used were Silencer Select siRNA specific for BRD4 (ID: s23901, Thermo Scientific, Waltham, MA, USA) and control Silencer Select RNAi Negative Control (Thermo Scientific, Waltham, MA, USA) at a final concentration of 20 nM.

For plasmids transfection: hCMV‐ORF‐N‐HA‐IRES‐puro for Homo sapiens DNM1L (or the empty vector) plasmid,^24^ was transfected with Lipofectamine2000 (Thermo Fisher Scientific) reagent according to the transfection manufacturer's protocol. Day after transfection, the cells were treated with DMSO (CNT) and BETi for 2 days.

### 2.7 RNA isolation and reverse‐transcription quantitative PCR assays

Total RNA was extracted using Maxwell RSC simplyRNA Cells (Promega) kit following the manufacturer's procedure. cDNA was synthesized from up to 1 μg of RNA using the iScript cDNA Kit (Bio‐Rad). Reverse‐transcription quantitative PCR (RT‐qPCR) was performed using goTaq qPCR Master Mix (Promega) and primers (Eurofins) mixed at a final concentration of 250 nmol/L in the CFX96 Real‐Time PCR Detection System (Bio‐Rad). The 2^(−ΔΔ*Ct*)^ method was used to calculate RNA expression, then results were expressed as fold change on respective control. βACTIN (b‐ACT) was used as a housekeeper. RT‐qPCR primers are listed in Table 1.

**TABLE 1:** List of antibodies, and expression primers for RT‐PCR.

Name	Antibody Information	
cMYC	# 9E10 (SC40)	SantaCruz BT
BCL2	Catalogue # 13–8800	Invitrogen
CytC	Cytochrome c (136F3) Rabbit mAb 4280	Cell Signalling
OPA1	OPA1 (D6U6N) Rabbit mAb 80,471	
MFN2	Mitofusin‐2 (D1E9) Rabbit mAb 11,925	
MFF	MFF (E5W4M) XP® Rabbit mAb 84,580	
pDRP1 (s616)	Phospho‐DRP1 (Ser616) (D9A1) Rabbit mAb 4494	
DRP1	DRP1 (D6C7) Rabbit mAb 8570	
SDHa	SDHA (D6J9M) XP® Rabbit mAb 11,998	
COX IV	COX IV (3E11) Rabbit mAb 4850	
PDHa	Pyruvate Dehydrogenase (C54G1) Rabbit mAb 3205	
ACC	Acetyl‐CoA Carboxylase (C83B10) Rabbit mAb	
SOD1	SOD1 (71G8) Mouse mAb 4266	
Tom20	Tom20 (D8T4N) Rabbit mAb 42,406	
ATP5a	Total OXPHOS Human WB Antibody Cocktail (ab110411)	Abcam
BRD4	# A301‐985A100	Bethyl
bACt	# A1978	Sigma‐Aldrich

Name	Forward	Reverse
DRP1	CAAAGCAGTTTGCCTGTGGA	TCTTGGAGGACTATGGCAGC
SDHA	AGGCTTGCGAGCTGCATTTG	AGCCCTTCACGGTGTCGTAG
bACT	TGCGTTACACCCTTTCTTGA	AAAGCCATGCCAATCTCATC

### 2.8 mtDNA copy number

Total DNA from BETi‐treated cells and BRD4 siRNA was obtained by Maxwell RSC blood DNA kit (Promega) extraction. 1 ng of genomic DNA was used to quantify mtDNA by RT‐qPCR. The mtDNA/nuclear DNA content was assessed using specific primers designed for the mitochondrial ND1 gene (for 5′‐CCCGCCACATCTACCATCA‐3′ and rev: 5′‐GAAGAGCGATGGTGAGAGCTAAG‐3′) and the nuclear GAPDH gene (for 5′‐CAATTCCCCATCTCAGTCGT‐3′ and rev: 5′‐GCAGCAGGACACTAGGGAGT‐3′). The ratio between these two genes (ND1/GAPDH) identifies the relative mtDNA content.

### 2.9 Metabolic flux analysis with the seahorse XFe96

Real‐time extracellular acidification rates (ECARs) and oxygen consumption rates (OCRs) were determined using the Seahorse Extracellular Flux (XFe96) analyzer (Agilent). Briefly, 2 × 10^4^ cells per well were seeded into XFe96 well cell culture plates and incubated for 24 h to allow cell attachment. After 24 h, cells were treated with JQ1 and OTX (1 μM respectively) for 24 h. Vehicle‐alone (DMSO) control cells were processed in parallel, then, washed in pre‐warmed XF assay media (or for OCR measurement, XF assay media supplemented with 10 mM glucose, 0.5 mM Pyruvate, and 2 mM l‐glutamine). Cells were then maintained in 175 μL/well of XF assay media at 37 °C, in a non‐CO_2_ incubator for 1 h. During the incubation time, we loaded 25 μL of 80 mM glucose, 9 μM oligomycin, and 0.5 M 2‐deoxyglucose (for ECAR measurement) or 10 μM oligomycin, 10 μM CCCP, 10 μM rotenone, 10 μM antimycin A (for OCR measurement), in XF assay media into the injection ports in the XFe96 sensor cartridge. Measurements were normalized by protein content (SRB assay).

### 2.10 Mitochondrial membrane potential measurement

Cells were incubated with 1 nM tetramethyl rhodamine methyl ester (TMRM, Invitrogen) at 37°C for 30 min in the presence of 1 μM of BET inhibitor or DMSO for the indicated time points. TMRM fluorescence was measured by flow cytometry (FACS Aria, BD Biosciences) for at least 10,000 events.

### 2.11 Western blot

Cells were lysed with PLB‐Passive Lysis Buffer (Promega) supplemented with Protease (Medchem) and Phosphatase Inhibitors cocktail (Thermo Fisher Scientific). 1–30 μg of total lysate were analysed by SDS–PAGE using Bio‐Rad apparatus (Bio‐Rad). Immunoblot detection was performed with the appropriate HRP‐conjugated secondary antibodies (GE Healthcare) and Clarity Western ECL substrate (Bio‐Rad) or WESTAR ηC ultra 2.0 (Cyanagen). See Table 1 for antibodies used. All western blots were performed 2–4 times. Representative images were used in the figures. Protein expression quantification was performed using ImageJ (Fiji) software.

### 2.12 Mitochondrial morphology (confocal microscopy)

After 24 h of BETi treatment, cells were stained with MitoTracker Red CMXRos (Thermo Fisher Scientific) for 30 min at 37°C in DMEM only and extensively washed with PBS and fixed in 4% Paraformaldehyde (PFA, Calbiochem) in PBS for 15 min at room temperature. Cells were permeabilized with 0.1% Triton (Merck Sigma‐Aldrich) in PBS and cell nuclei stained by using Hoechst 33342 (2′‐[4‐ethoxyphenyl]‐5‐[4‐methyl‐1‐piperazinyl]‐2,5′‐bi‐1H‐benzimidazole trihydrochloride trihydrate) Solution (Thermo Fisher Scientific). Immunofluorescence images were acquired with a TCS SP8 laser scanning confocal microscope (Leica Microsystems, Wetzlar, Germany). Acquisition parameters were kept constant in all the experiment conditions. At least *n* = 5 images have been acquired for each condition and processed on Zeiss as well as Metamorph software.

### 2.13 Transmission electron microscopy (TEM)

Samples were processed according to standard protocols for ultrastructural TEM analysis. Cell pellets were fixed in 3% glutaraldehyde (Merck Sigma‐Aldrich) in 0.1 M phosphate buffer at pH 7.4 (ClinicalSciences) for 2 h at 4°C. After 2 h, three washings were carried out in phosphate buffer at 4°C to eliminate any residual fixative. A post‐fixation in 1% osmium tetroxide in phosphate buffer (0.1 M pH 7.4) was then performed for 2 h at 4°C to preserve the lipid structures. Samples were washed 3 times in phosphate buffer, subjected to gradual dehydration using increasing concentrations of acetone, and embedded with epoxy resin (Epon). Ultrathin sections (60–90 nm) were obtained using an RMC PowerTome series ultramicrotome with a Diatome diamond knife, collected on 300 mesh copper grids, and observed with a Jeol JEM‐1400 Plus transmission electron microscope operating at 80 kV. Statistical significance was verified using the lengths of 20 mitochondria per cell. At least 30 cells were counted for each condition. Data (presented as mean ± S.E.M.) were evaluated by the one‐way analysis of variance (ANOVA) and Tukey's test.

### 2.14 Nuclear magnetic resonance (NMR) spectroscopy analysis

Aqueous and organic metabolites were extracted from MDA‐MB231 and Hs578t, and treated for 24 h with BETi according to the protocol previously described.^4^ The polar phase containing water‐soluble cellular metabolites was evaporated using a rotary evaporator and lyophilized while the organic fraction (lipid phase) was evaporated under nitrogen gas flow. Both phases of cell extracts were stored at −20°C. The aqueous fraction from cells and extracellular medium samples (300 μL) were reconstituted in 700 μL D2O using TSP (0.1 mM) as NMR internal standard. High‐resolution 1H‐NMR analyses were performed at 25°C at 14.1 T Bruker AVANCE Neo spectrometer (Karlsruhe, Germany, Europe) on aqueous cell extracts using acquisition pulses (noesypr1d pulse sequence for water suppression), data processing, and peak area deconvolution as previously described.^26^ The absolute quantification of aqueous metabolites, determined by comparing the integral of each metabolite to the integral of reference standard TSP and corrected by respective proton numbers for metabolite and TSP, was expressed as nmoles/10^6^ cells and then converted into metabolite percentage (relative to total metabolites evaluated in each sample). *Reagents*: deuterated reagents (methanol (CD3OD), chloroform (CDCl3), and deuterium oxide (D2O) were purchased from Cambridge Isotope Laboratories, Inc.; 3‐(trimethylsilyl)propionic‐2,2,3,3‐d4 acid sodium salt (TSP) was obtained from Merck & Co, Montreal, Canada).

### 2.15 Statistical analysis

All the experiments were replicated two to five times. Statistical significance was calculated using an unpaired two‐tailed student's *t*‐test (GraphPad Prism 7) of the group drugs‐treated/siBRD4 versus the group control‐treated/siCNT. *p*‐values of less than 0.05 were considered significant. *p*‐values <0.05 are indicated with * or #, *p*‐values <0.01 are indicated with ** or ##, and *p*‐values <0.001 are indicated with *** or ###. All data represent the mean ± S.E.M. (*n* ≥3). For the Seahorse analyses, data sets were analysed using XF software and GraphPad Prism software, using two‐way ANOVA and Student's *t‐*test calculations. All experiments were performed in quintuplicate, three times independently. Data represent the % average ± SEM over control cells (CTN), *n* = 3, *p < 0.05, **p < 0.005, ***p < 0.0005, ****p < 0.00005. Graphical abstract and schematic representations were created with BioRender.com.

## 3 RESULTS

### 3.1 BETi promoted cell death through the mitochondrial pathway in vitro

**FIGURE 1:**
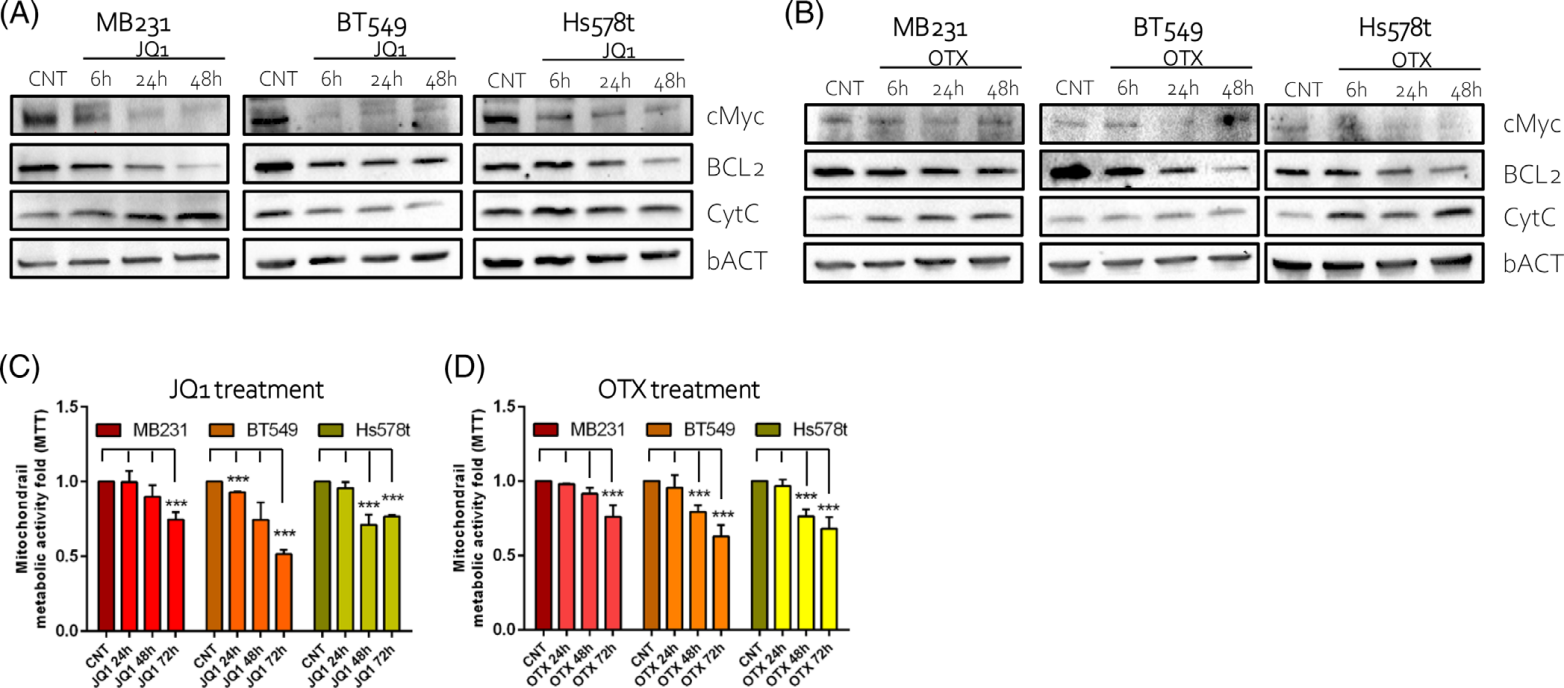
BET inhibitors (BETi) promoted cell death through the mitochondrial pathway in vitro. (A), (B). Western blot images of TNBC cells treated with JQ1 (A) and OTX015 (OTX, B) over 2 days compared to control (CNT). Blots are probed with antibodies against cMyc, BCL‐2, CytC, and β‐Actin (bACT). (C), (D). MTT assay on TNBC cells treated with JQ1 (C) and OTX (D) over 3 days compared to control (CNT). The results are presented as a fold change of metabolic viability normalized to the relative control (CNT). BETi were used at a final concentration of 1 μM.

We recently demonstrated that BETi induces cell cycle arrest and cell death by upregulating p21 expression and by altering mitochondria dynamics in TNBC cell lines^4^ (Supplementary Figure 1A). To establish to which extent mitochondria perturbations partake to the pro‐apoptotic effects of BETi, MDA‐MB231, BT549, and Hs578t were treated with two different BETi (JQ1 and OTX) and evaluated the protein expression of BCL‐2 and cytochrome C (CytC), two apoptotic markers. We also assessed cMyc expression. cMyc is one of the primary targets of BRD4^27^ and a cooperator of the mitochondrial apoptotic pathway mediated by BCL‐2, an established target of BRD4.^22^, ^28^, ^29^ The expression of cMyc and BCL‐2 decreased over time after treatment, while CytC expression was induced (Figure 1A, B and quantified in Supplementary Figure 1B, C). The different changes occurring upon treatments and timing likely reflected an altered dynamism that mirrored the transcriptional and metabolic heterogeneity of the TNBC cells.^30^ To further correlate the effect of these inhibitors with mitochondria function, we measured by MTT assay^31^ the mitochondrial metabolic activity after BETi treatment. Consistently, mitochondrial metabolic activity decreased over time (Figure 1C, D). Overall, these results suggested that the pro‐death effects of BETi involved the mitochondrial pathway and the cMyc‐BCL2 axis. Mitochondria are a major site of ROS generation and excess levels of ROS may induce cell death.^32^, ^33^ TNBC cells have an elevated oxidative state when compared with non‐tumorigenic or luminal BC cells.^34^ After 24 h of BETi administration, the ROS levels were almost unaltered in MB231 and Hs578t cells as well as the expression of superoxide dismutase (SOD1), one of the major antioxidant enzymes, Instead, BT549 cells decreased ROS levels and upregulated SOD1 expression (Supplementary Figure 1D, E). This data suggested that BETi‐induced cell death is not fully associated with ROS production.

### 3.2 BETi alter the mitochondrial fission/fusion equilibrium and increase cell death

**FIGURE 2:**
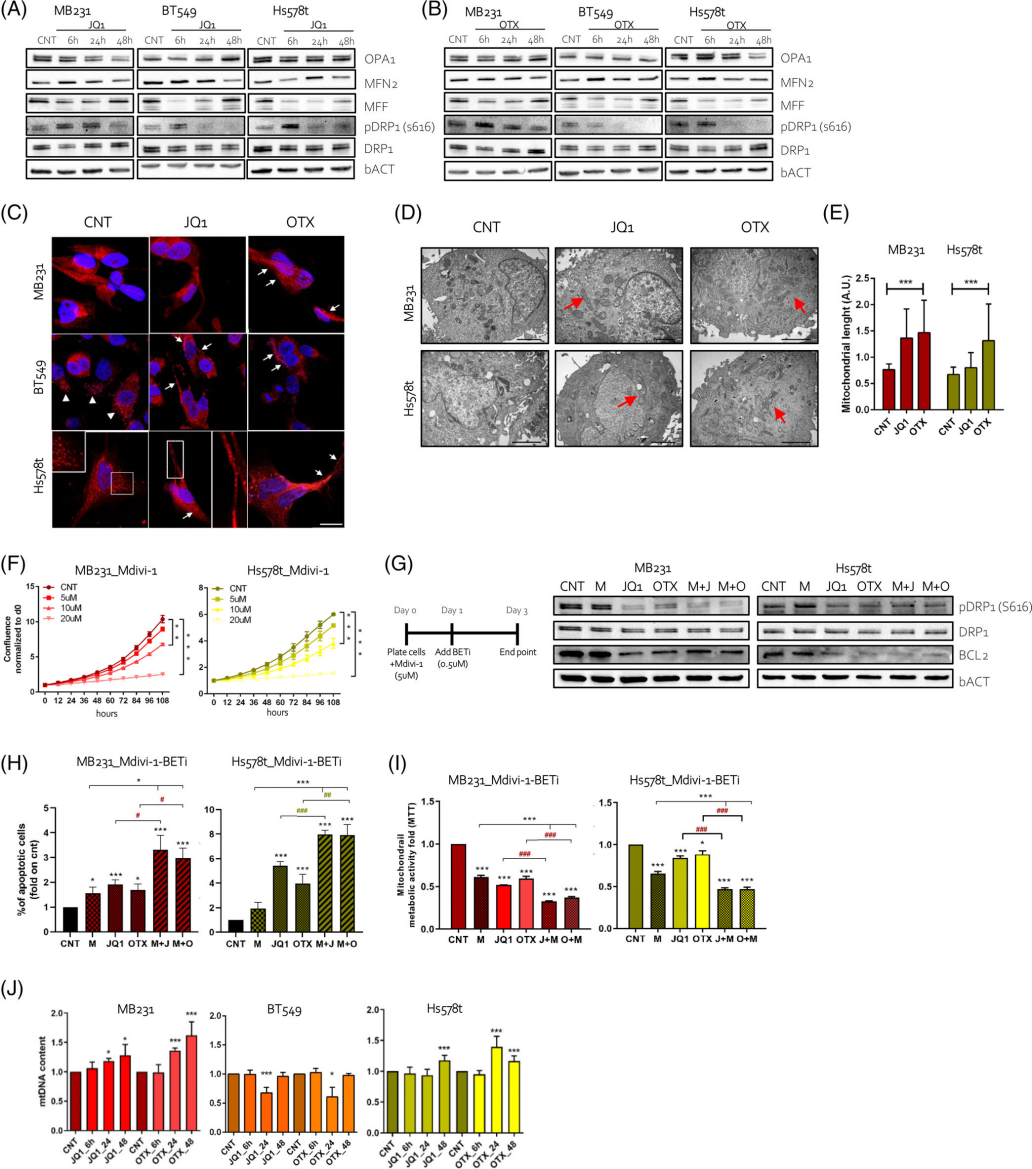
BETi administration impaired mitochondria fission. (A), (B) Western blot images of TNBC cells treated with JQ1 (A) and OTX (B) over 2 days compared to control (CNT). Blots are probed with antibodies against OPA1, MFN2, MFF, pDRP1 (S616), DRP1, and β‐Actin (bACT). (C) Representative confocal images of mitochondrial morphology in TNBC cells treated for 24 h with JQ1 and OTX and stained for Mitotracker Red and Hoechst. Scale bars: 20 μm. (D) Representative TEM images of MB231 and Hs578t cells treated for 24 h with JQ1 and OTX. Scale bar: 2 μm, except for MB231 treated with JQ1, in which the scale bar is 1 μm. CNT MB231: Original magnification ×5000; CNT Hs578t: Original magnification ×6000; JQ1 MB231: Original magnification ×8000; JQ1 Hs578t: Original magnification ×5000; OTX MB231: Original magnification ×6000; OTX Hs578t: Original magnification ×5000. (E) Quantification of the mitochondrial length of the experiment in panel (D). Data (presented as mean ± S.E.M.) were evaluated by the one‐way analysis of variance (ANOVA) and Tukey's test. (F) Cell proliferation assay was measured by IncuCyte analysis to observe the effect of different concentrations (5–10‐20 μM) of Mdivi‐1 on TNBC cells over 108 h of treatment. (G) On the left, a schematic timeline of the treatment. On the right, western blot images of MB231 and Hs578t cells treated with Mdivi‐1 and BETi compared to control (CNT). Blots are probed with antibodies against pDRP1 (S616), DRP1, BCL2, and β‐Actin (bACT). (H) Percentage of apoptotic cells in MB231 and Hs578t cells treated with Mdivi‐1 (M) and/or with BETi (JQ1, J and OTX, O) with Annexin V staining. (I) MTT assay to assess the combined effect of Mdivi‐1 (M) and/or with BETi (JQ1, J and OTX, O) on mitochondrial metabolic activity. The results in (H) and (I) are presented as a fold to the relative control (CNT) and the significance is calculated versus the CNT‐treated cell (*, ***) and BETi versus Mdivi‐1 + BETi (#, ##, ###). (J) The mtDNA content quantification of TNBC cells treated with JQ1 and OTX over 2 days of treatment. The results are presented as a fold change of treated cells to the relative control (CNT). (A)–(E), (J). BETi were used at a final concentration of 1 μM. (G)–(I) Mdivi‐1 and BETi were used at a final concentration of 5 and 0.5 μM, respectively.

Prompted by these results, we evaluated the impact of BETi on mitochondrial fission/fusion balance. Thus, we analysed the protein expression of the canonical mitochondrial fission or fusion markers OPA1 (OPA1 Mitochondrial Dynamin Like GTPase), MFN2 (Mitofusin 2), MFF (Mitochondrial fission factor), and DNM1L (Dynamin 1 Like, which encodes for DRP1) and the DRP1 phosphorylation at Ser616 (pDRP1), which enhances DPR1 activity, the primary effector of mitochondrial fission.^35^ To this end, TNBC cells treated for 6, 24, and 48 h with both BETi resulted in a consistent reduction of pDRP1. As well, the expression of MFF was reduced after exposure to both drugs. Conversely, the expression of OPA1 and MFN2, fusion‐associated proteins, did not display consistent alterations, overall indicating a potential reduction of mitochondrial fission (Figure 2A, B, quantified in Supplementary Figure 1F–H), further confirmed by immunofluorescence and TEM. MitoTracker immunostaining in control cells revealed a mitochondria morphology characterized by a mixed reticulum with tubular and round forms (or fragmentation) (white arrowhead, Figure 2C). By contrast, an elongated mitochondrial network, (white arrows, Figure 2C), was observed in TNBC cells upon BETi (comparing JQ1 and OTX vs. relative controls), following a potential reduction of the mitochondria fission. TEM confirmed an increase of elongated mitochondria in BETi‐treated cells (Figure 2D and quantified in Figure 2E). To assess whether the inhibition of fission is a crucial mediator of the BETi, we treated the cells with Mdivi‐1, a specific pharmacological inhibitor of the DRP1 activity.^36^ Mdivi‐1 treatment significantly affected cell growth similarly to BETi (Figure 2F and Supplementary Figure 1I) suggesting that mitochondrial fission is necessary for TNBC growth. To this end, first, we investigated the effect of Mdivi‐1 treated or cotreated with BETi on the phosphorylation of Drp1 (S616) on MB231 and Hs578t cells. Under these drug conditions (Figure 2G, left), the effect of Mdivi‐1 on the phosphorylation of DRP1 (Ser616) was unaffected, while we confirmed the marked inhibition of BETi on this residue. Similarly, the expression of BCL2 was downregulated only after BETi administration (Figure 2G, right). In terms of apoptosis and mitochondrial metabolic activity (MTT), Mdivi‐1 treatment alone induced cell death only in MB231 and reduced mitochondrial metabolic activity comparable to JQ1 and OTX alone (Figure 2H, I) in both TNBC cells. When BETi was added to pretreated Mdivi‐1 cells the fractions of unviable cells were significantly increased to the BETi‐treatment alone (Figure 2H), and the mitochondrial metabolic activity was significantly reduced in Mdivi‐1‐BETi treated cells (Figure 2I). Conversely, the over‐expression of DRP1 (Supplementary Figure 1J) was not sufficient to antagonize BETi to induce cell death (Supplementary Figure 1K), suggesting that DRP1 alone is not the only mediator of this effect. We also observed a significant increase of mtDNA content in MB231 and Hs578t, a perturbation of the mtDNA content in BT549 cells only at 24 h, and unaltered mtDNA content in MCF7 upon treatment with BETi (Figure 2J and Supplementary Figure 1L). Interestingly, we found that the expression of Tom20, a reliable marker of the mitochondrial mass was downregulated after BETi exposure (Supplementary Figure 1M). Taken together, our findings showed that BETi altered mitochondrial fission/fusion dynamics, and mtDNA content and reduced the mitochondrial mass, which contribute to cell death.

### 3.3 BETi treatment provokes metabolic reprogramming decreasing glycolysis and altering OXPHOS metabolism

**FIGURE 3:**
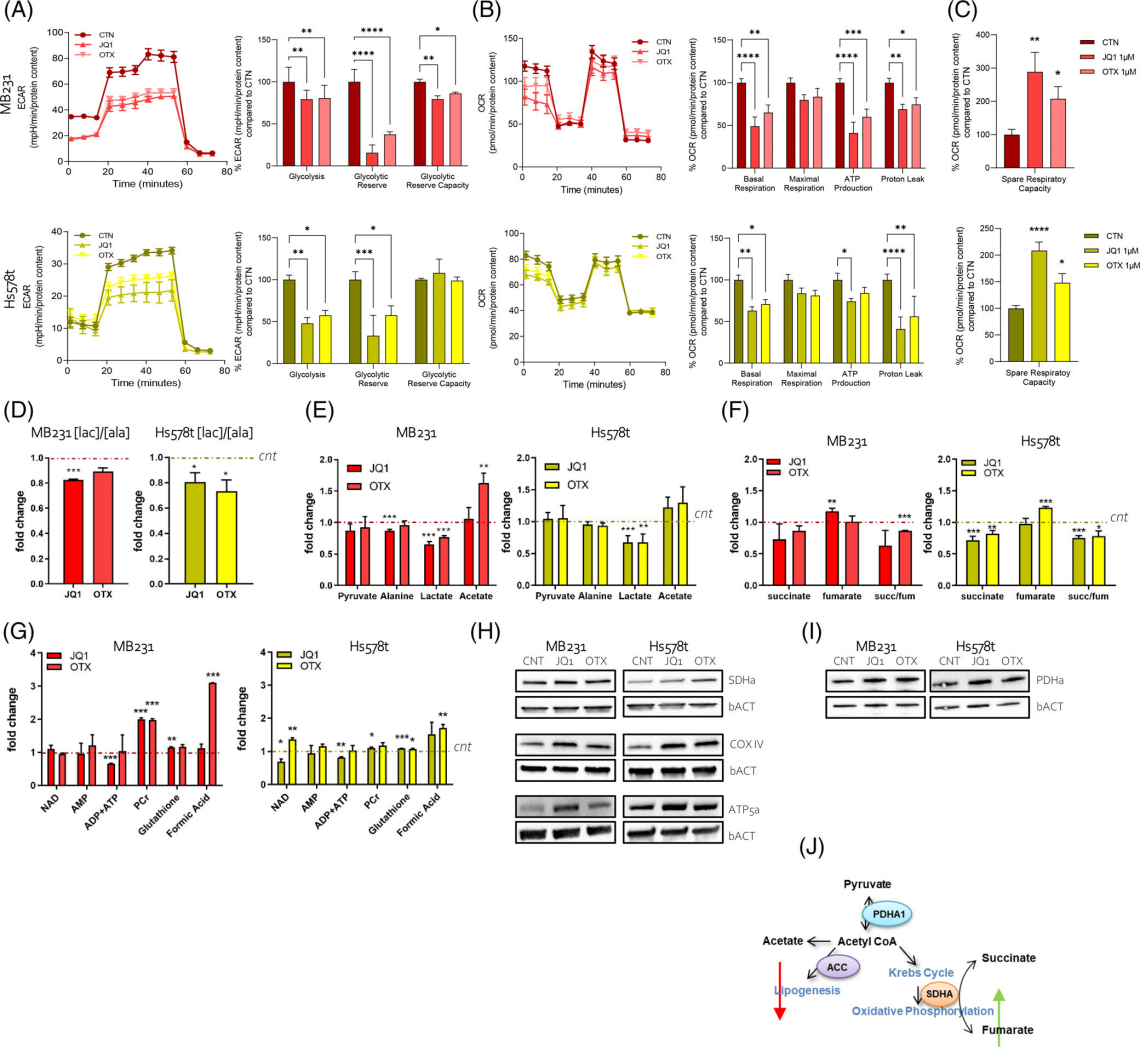
BETi provoked important metabolic variation. The extracellular acidification rate (ECAR) and the oxygen consumption rate (OCR) were determined using the Seahorse XFe96, via metabolic flux analysis. (A), (B) At the top, the MDA‐MB‐231 cell population treated (24 h) with JQ1 and OTX, showed a decrease in glycolysis, glycolytic reserve, and glycolytic reserve capacity, as well as a decrease in basal respiration, mitochondrial ATP‐production, and proton leak after treatment with both compounds. At the bottom, the Hs578t cell population treated (24 h) with JQ1 and OTX, showed a decrease in glycolysis, and glycolytic reserve, as well as a decrease in basal respiration, and proton leak after treatment with both compounds. Mitochondrial ATP production level was reduced after treatment with the JQ1 compound. (C) Spare respiratory capacity level was observed to increase after 24 h treatment with JQ1 and OTX in both cell lines (MDA.MB‐231 and Hs578t) Cell populations were analysed 24 h after plating. (D) NMR analysis of the concentration of lactate and alanine in the supernatant of MB231 and Hs578t cells treated with JQ1 and OTX for 24 h and expressed as a ratio ([lac]/[ala]). (E), (F) NMR measurement of the intracellular levels of pyruvate, alanine, lactate, and acetate (E) and succinate and fumarate and ratio between them (succ/fum) (F) in MB231 and Hs578t cells treated with JQ1 and OTX for 24 h. (G) NMR analysis of the intracellular concentration of NAD, AMP, ADP + ATP, phosphocreatine (PCr), glutathione, and formic acid in MB231 and Hs578t cells after 24 h of BETi treatment. The results in figure (D)–(G) are presented as a fold change to the relative control (cnt). (H) Western blot images of TNBC cells treated with JQ1 and OTX for 24 h compared to control (CNT). Blots are probed with antibodies against SDHa, COX IV, ATP5a, and β‐Actin (bACT). (I) Western blot images of TNBC cells treated with BETi for 24 h compared to control (CNT). Blots are probed with antibodies against PDHa and β‐Actin (bACT). (J) Graphical explanation of metabolic pathways associated with the switch from lipogenesis to oxidative phosphorylation. BETi were used at a final concentration of 1 μM.

We first evaluated the mitochondrial bioenergetics profiles of TNBC cells treated for 24 h with BETi by measuring their OCR and ECAR. A Seahorse XFe96 was used to measure the ECAR and OCR of each cell line, as the indicators of pyruvate‐lactate production during glycolysis and mitochondrial respiration during OXPHOS, respectively. Upon BETi treatment, both TNBC cell lines exhibited a significant decrease in glycolytic capacity (Figure 3A) and reduced basal respiration and ATP production (Figure 3B) compared with the control‐treated cells (CNT). Through, both TNBC cells shared a significant spare respiratory capacity (Figure 3C) indicating that the cells are still capable of responding to an energetic demand. Ultimately, we wondered whether mitochondrial alteration results in metabolic alterations. Thus, we performed NMR with the intent to confirm extra and intra‐metabolome variations. First, we estimate the glycolysis/OXPHOS balance in the supernatant based on the lactate/alanine (lac/ala) ratio. This ratio is associated with a cellular redox state due to the conversion of pyruvate to lactate and alanine coupled with NADH /NAD+ ratio (as suggested by Petrella et al.^37^). We confirmed the significant decrease in the lac/ala ratio in BETi‐treated MB231 and Hs578t cells (Figure 3D). Intracellularly, the levels of pyruvate are stable, the levels of lactate, and slightly alanine, decrease, while the levels of acetate increase (Figure 3E). The changes in these metabolites suggest a shift of pyruvate metabolism towards acetate and, consequently, an increase in acetyl‐CoA synthesis and OXPHOS metabolism. These results follow the decrease of glycolysis (Figure 3D). We also quantified the differences in succinate and fumarate concentrations in MB231 and Hs578t after BETi administration. BETi treatment reduces the levels of succinate compared with CTN cells. By contrast, the level of fumarate increased upon treatment thus determining a significant reduction of succinate/fumarate ratio (Figure 3F). All together, these data indicated that BETi alters glycolysis metabolism and metabolic pathways in favour of OXPHOS metabolism. In line, we also found that BETi induced an increase of intracellular phosphocreatine (PCr) and formic acid levels, a variable perturbation in NAD and AMP/ADP ratio and ATP levels (Figure 3G), and alterations in amino acids quantities (Supplementary Figure 2A, B). Generally, amino acid metabolic pathways are associated with dysfunctional mitochondria. Interestingly, significant accumulation of glutathione, an important regulator of cellular redox state in protecting cells from damage caused by ROS,^38^ is in accordance with the ROS levels after BETi administration (see Supplementary Figure 1D). We also evaluated the expression levels of respiratory enzymes of the mitochondrial respiratory chain such as SDHa (complex II), COX IV (complex IV), and ATP5a (complex V) in both MB231 and Hs578t cells. BETi treatment led to an increase of these proteins (Figure 3H); this agrees with the observed increase expression of Cytc (Figure 1A, B). We also detected an increase of PDHa (the main active subunit of the pyruvate dehydrogenase complex, PDH) (Figure 3I) and a decrease in acetyl‐CoA carboxylase (ACC) level in BETi‐treated cells (Supplementary Figure 2C), indicating that BETi also negatively affected lipogenesis (pathways graphically described in Figure 3J). Finally, we confirmed changes in SDHa, PDHa, and ACC protein levels (Supplementary Figure 2D) in BT549 cells. All these data evidenced that BETi drastically reduced glycolysis and influenced OXPHOS metabolism by affecting mitochondrial dynamics.

### 3.4 BRD4 alters mitochondrial dynamics by modulating DRP1 expression

**FIGURE 4:**
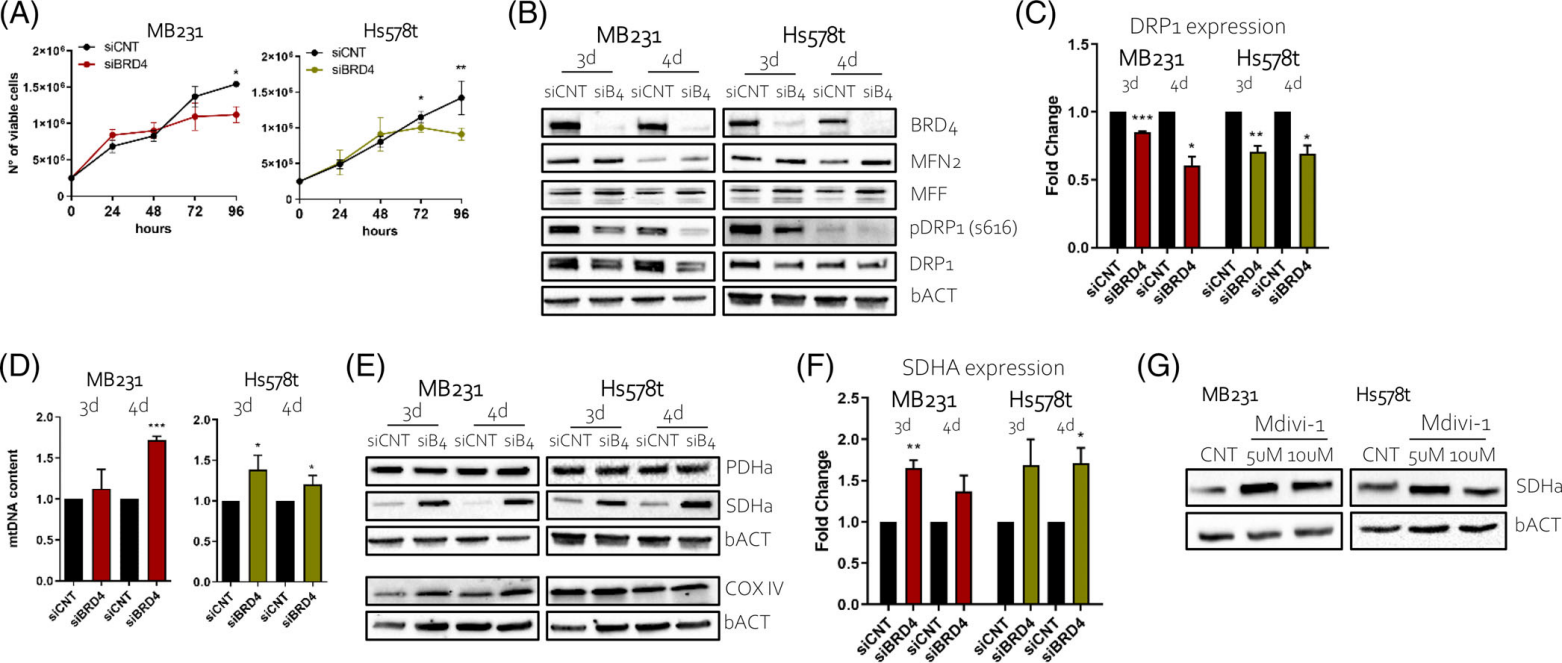
BRD4 affected the proliferation and mitochondria dynamics of TNBC cells. (A) Cell proliferation assay measured by Trypan Blue after siRNA transfection of BRD4 (siBRD4) or control (siCNT) on MB231 and Hs578t overtime. (B) Western blot images of MB231 and Hs578t cells after transfection of control (siCNT) or siBRD4 (siB4) at day 3 (3 d) and day 4 (4 d). Blots are probed with antibodies against BRD4, MFN2, MFF, pDRP1 (s616), DRP1, and β‐Actin (bACT). (C) qRT‐PCR analysis of DRP1 expression in MB231 and HS578t after 3 and 4 days (3 d and 4 d, respectively) of siBRD4 transfection compared to siCNT. (D) The mtDNA content quantification of MB231 and Hs578t cells after 3 (3 d) and 4 (4 d) days of siCNT and siBRD4 transfection. (E) Western blot images of MB231 and Hs578t cells after transfection of control (siCNT) or siBRD4 (siB4) at day 3 (3 d) and day 4 (4 d). Blots are probed with antibodies against PDHa, SDHa, COX IV, and β‐Actin (bACT). (F) qRT‐PCR analysis of SDHa expression in MB231 and HS578t after 3 and 4 days (3 d and 4 d, respectively) of siBRD4 transfection compared to siCNT. (G) Western blot images of TNBC cells treated with 5 and 10 μM of Mdivi‐1 for 24 h compared to control (CNT). Blots are probed with antibodies against SDHa and β‐Actin (bACT).

BETi targets all members of the BET family. However, inhibition of BRD4 is recognized as the most prominent effector of their action in cancer, due to the high dependency of cancer cells on the transcriptional activity of this factor. To assess if the mitochondrial alterations observed with the drugs were mediated by BRD4 inhibition, we silenced BRD4 by siRNA (siBRD4) in TNBC cells and measured to which extent its targeting phenocopied the effects of BETi. siBRD4 affected cellular proliferation (Figure 4A) and BCL2 expression (Supplementary Figure 2E) like BETi treatment. Silencing of BRD4 also reduced pDRP1 (Ser616) levels and slightly increased MFF in HS578t cells (Figure 4B and quantified in Supplementary Figure 2F). siBRD4, as well, caused a significant downregulation of DRP1 mRNA (Figure 4C). Likewise to BETi, after siBRD4 transfection, the mtDNA content increased (Figure 4D). Thus, we evaluated the effect of BRD4 silencing on the expression of SDHa, PDHa, and COX IV. Interestingly, siBRD4 enhanced the expression of SDHa both at protein and mRNA levels (Figure 4E, F) but did not affect the expression level of PDHa or COX IV (Figure 4E), confirming that both the inhibition and the genetic targeting of BRD4 push the cells towards OXPHOS metabolism. Remarkably, Mdivi‐1 treatment increased the SDHa expression (Figure 4G and Supplementary Figure 2G), again suggesting that mitochondrial fission driven by BET proteins might represent a valuable therapeutic target in breast cancer.

### 3.5 Metformin and BETi drug combinations are additive in TNBC cells

**FIGURE 5:**
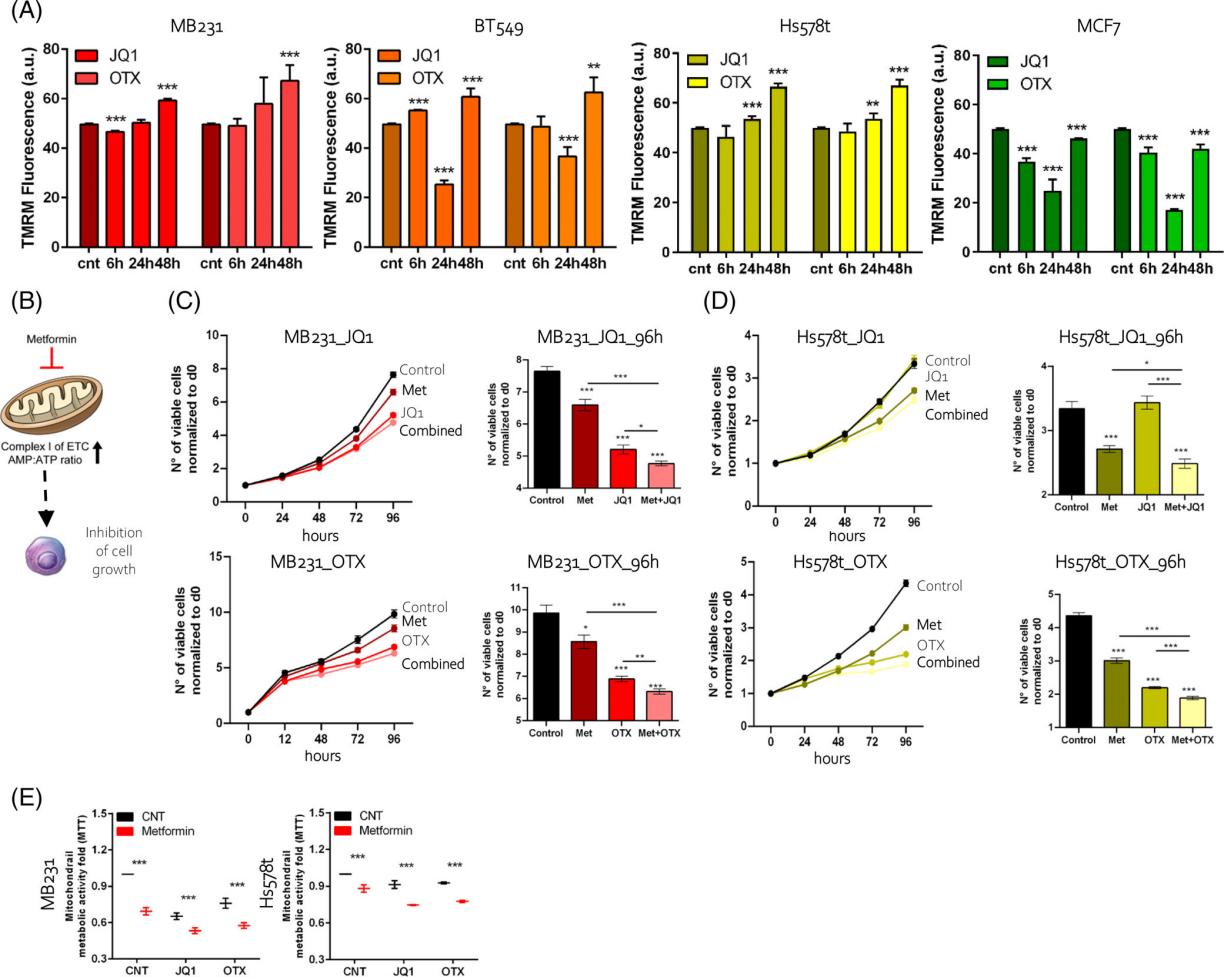
BETi and Metformin reduced the growth and metabolic activity of TNBC cells. (A) Quantitative analysis of mitochondrial TMRM fluorescence in BC cells treated with JQ1 and OTX over a 2‐day time compared to control (CNT). (B) Schematic representation of the mechanism of cell growth inhibition by Metformin (Met). (C) Cell proliferation assay measured by IncuCyte of MB231 treated with JQ1 (0.1 μM, top) and OTX (0.2 μM, bottom) in combination with Met (0.5 mM) over 4 days of treatment (96 h). (D) Cell proliferation assay measured by IncuCyte of Hs578t treated with JQ1 (0.02 μM, top) and OTX (0.5 μM, bottom) in combination with Met (1 mM) over 4 days of treatment (96 h). At the right side of each graph there are the number of viable cells counted at 96 h of treatments. (E) MB231 and Hs578t cells treated with BETi (0.2 μM JQ1 and OTX) alone or in combination with Metformin (5 mM for MB231 and 1 mM for HS578t) for 3 days compared to control (CNT). The MTT results are presented as a fold change of metabolic viability normalized to the relative control (CNT).

Mitochondria membranes are characterized by a potential (∆Ψm) generated by protons released by complexes I, III, and IV of the ETC during OXPHOS transformations associated with the activity of the Krebs cycle.^39^, ^40^ Thus, we conducted ∆Ψm measuring in BETi‐treated and CTN cells observing, as expected, a significant increase of this parameter after BETi exposure in MDA‐MB231 and Hs578t cells. In BT549, ∆Ψm potential showed a transient drop at 24 h of JQ1 and OTX administration and an increase later during the treatment. Instead, the increase was not observed in MCF7 cells (Figure 5A). To prove that the anticancer potential of BETi is attributed to the enhanced OXPHOS phenotype, we used Metformin (Met) in combination with BETi. It is reported that Met treatment decreases the intracellular levels of several metabolites in the TCA cycle, including α‐ketoglutarate (α‐KG) and succinate, and induces a shift towards glycolysis (graphically described in Figure 5B).^41^, ^42^, ^43^ In our experimental conditions, MB231 and Hs578t cell lines treated with a sub‐lethal dose of Met markedly shifted to glycolysis as measured by the extracellular lac/ala ratio (data not shown). Thus, we hypothesized that the addition of Met to BETi‐treated cells could be synergistic in reducing cell growth. To test this, we exposed MB231 (Figure 5C) and Hs578t (Figure 5D) cells to BETi as single agents or in combination with Met for 4 days with different doses (Figure 5C, D). We also calculated the combinatorial index (C.I.) of these drugs. BETi (JQ1 0.1 μM and 0.02 μM, OTX 0.2 μM and 0.5 μM in MB231 and HS578t, respectively) and Met (0.5 and 1 mM in MB231 and HS578t, respectively) C.I. values are 1 for JQ1 and 0.7 for OTX in MB231 and 0.9 for JQ1 and 0.9 for OTX in HS578t. This indicates that the combination of BETi and Met achieved additive, and not synergistic, cytotoxic effects. Accordingly, the mitochondrial metabolic activity measured by MTT assay was significantly reduced in both single or combined‐treated cells (BETi+Met, red) compared with the BETi treated only (BETi in black, Figure 5E). All together, these data indicate that the in vitro anti‐tumoral effects of BETi are derived from an alteration of the mitochondrial dynamic metabolisms.

## 4 CONCLUSIONS

Over 20 clinical trials are underway evaluating the anti‐cancer properties of BETi for treating human malignancies, including TNBC. Unfortunately, significant toxicities, most of which are dose‐dependent, have been observed in phase I trials.^44^, ^45^, ^46^ The relatively narrow therapeutic window jeopardizes the prospect of subsequent BETi clinical trials and the ultimate implementation of these drugs in patient care, especially for TNBC patients with limited therapeutic options. BETi exert anti‐tumour functions by blocking cell proliferation, inducing cell death, and inhibiting angiogenesis.^4^, ^47^, ^48^ We recently demonstrated that in TNBC, the enzymatic activity of ATGL could help BETi to reduce the mitochondrial metabolic activity and induce mitochondria oxidative stress leading to cell death.^4^ From those pieces of evidence, we look forward to exploring how and to which extent BETi alter mitochondria biology and how these alterations may contribute to the cytotoxic effects of these drugs. Here, we confirmed that two different BETi (JQ1 and OTX015) affect mitochondrial pathways suppressing cMyc and BCL‐2 expression, upregulating CytC expression, and altering metabolic profiles. Mitochondria are an important research focus: they play a central role in cellular energy metabolism, apoptosis, and multiple cellular signaling pathways.^49^ Biogenesis and turnover of mitochondria, fission, and fusion dynamics, oxidative stress regulation, metabolism, bioenergetics, and cell death regulation are characteristics of altered mitochondrial functions that control tumorigenesis, progression, and resistance to therapy.^50^, ^51^ In our study, we observed that BETi administration reduced mitochondrial fission, affecting mitochondria structure that, under drug exposure, resulted in a more elongated and organized network. The decreased expression of pDRP1 (Ser616) and MFF‐mediated in concomitance to the unaffected MNF2 and OPA1 expression caused an impairment of mitochondrial fission. TEM and confocal microscopy confirmed the increased mitochondrial length, which was consistent with the essential role of DRP1 for most types of mitochondrial fission. We also observed that these structural changes were associated with a specific metabolism rewiring which in turn represents a well‐established hallmark of cancer in TNBC as in other settings.^52^, ^53^, ^54^ Like most other cancers, TNBC relies on increased glucose uptake and glycolysis, making it a potential therapeutic target, although effective treatments curtailing glycolytic metabolism have not yet reached clinical practice.^55^ TNBC can rely also on oxidative metabolism (OXPHOS)^56^ promoting TNBC migration and metastasis dissemination.^57^ Changes in mitochondrial dynamics have been reported to affect OXPHOS by improving coupling efficiency or increasing mitochondrial mass.^58^ We observed that in TNBC cells, treatment with BETi alter OXPHOS by increasing the expression of respiratory enzymes without affecting mitochondrial mass. The impact of BETi on the OXPHOS phenotype was evidenced also by a significant increase in mtDNA content and ∆Ψm. Since all the TNBC cell lines tested grown under identical conditions, we hypothesized that the differences observed in BT549 with respect to MB231 and Hs578t might be related to genetic (i.e., EMT pathways) and/or metabolic differences.^59^ We also reported that the non‐TNBC MCF7 cells exposed to BETi do not show an increase in either mtDNA or ∆Ψm, suggesting that the reported mitochondrial alterations can be specific (or prominent) in TNBC rather than other BC subtypes. Lately, several studies indicated that the preferential sensitivity of TNBC cells to BETi compared to other BC lines likely arises from a combination of their dependency on BET proteins for survival and proliferation, specific gene expression patterns, and oncogenic dependencies. For example, the TNBC cells have distinct gene expression profiles exhibiting higher expression levels of genes regulated by BET proteins and often exhibit specific oncogenic dependencies that make them susceptible to targeted therapies. Since BET proteins regulate the expression of genes involved in oncogenic pathways, inhibition of BET proteins may disrupt these pathways more effectively in TNBC cells compared to other BC subtypes, and others.^60^, ^61^, ^62^ Moreover, from a metabolic point of view, BETi strongly reduced glycolysis, and dropped the lactate levels and lipogenesis by reducing ACC expression. The “switch‐off” of glycolysis and lipogenesis forced the acetyl‐CoA to enter into mitochondria altering the Krebs cycle in favour of OXPHOS. This is due to the increase of PDHa, SDHa, COX‐IV, and ATP5a expression. Nevertheless, in addition to its role in OXPHOS, SDH is a component of the TCA cycle, making a functional link between these two essential processes.^63^, ^64^, ^65^, ^66^, ^67^ A role for BRD4 in regulating the mitochondria dynamics has been elucidated in some solid tumours, but not in BC.^23^, ^68^ As a downstream target gene of BRD4, cMyc may be involved in the relationship between BRD4 and mitochondria dynamics. MFF, one of the DRP1 receptors, is a novel transcriptional target of cMyc.^69^ By modulating the expression of BRD4, we confirmed the downregulation of pDRP1 (Ser616), the increase in mtDNA content, and the upregulation of SDHa, which is consistent with BRD4 inhibition. We also used Mdivi‐1, a small molecule compound for DRP1‐mediated mitochondrial fission inhibition,^70^ to phenocopy the BRD4 silencing and BET function inhibition. We found that Mdivi‐1 treatment significantly reduced cell proliferation and induced the expression of SDHa as demonstrated for BETi or siBRD4. Interestingly, we demonstrated that the BETi‐induced cell death derived also from altered mitochondrial dynamics. TNBC cells with already fused mitochondria (treated with Mdivi‐1) are more prone to BETi‐cell death, and this has not been observed, as far as our knowledge, in breast cancer. However, further studies are needed to evaluate the network of mitochondrial shaping proteins likely involved in such anti‐tumour activity. The usage of BETi in clinical settings is still limited, however (1) targeting both pathways simultaneously could offer therapeutic benefits in TNBC treatment distinct from cytotoxic chemotherapy or unlimited the usage of BETi, and (2) BETi can offer potential target(s) to modulate mitochondrial respiration without directly hitting the OXPHOS. Inhibition of mitochondrial respiration is not a reliable therapeutic strategy for chemo‐resistant TNBC, given the predicted severe side effects of OXPHOS inhibition. Finally, the usage of JQ1 or OTX in combination with Metformin, a drug commonly used in clinical for patients with type‐2 diabetes mellitus that exerts anti‐tumoral effects in vitro and in vivo,^71^, ^72^ demonstrated an additive behaviour in reducing proliferation and mitochondrial metabolic activity of TNBC cells better than BETi as a single agent. This suggests that the combination of BETi with other drugs cannot only enhance the efficacy but also reduce the side effects of the single‐drug treatment.^73^, ^74^ In conclusion, pharmacological BRD4‐targeting by BETi affects mitochondrial dynamics, metabolism, and mtDNA content peculiar to BC cell proliferation. These aspects underscore the importance of novel mitochondrial pathways, which can help to develop targeted therapeutics against BC, especially in TNBC.

## AUTHOR CONTRIBUTIONS

**Teresa Rossi:** Conceptualization, methodology, validation, formal analysis, Investigation, writing‐original draft, writing‐reviewing and editing, project administration. **Egidio Iorio, Mattea Chirico, and Maria Elena Pisanu:** Validation, formal analysis, resources, data curation, writing‐reviewing, and editing. **Nicola Amodio:** Validation, resources, writing‐reviewing and editing, funding acquisition. **Maria Eugenia Gallo Cantafio and Ida Perrotta:** Validation, resources, formal analysis.**Francesca Colciaghi:** Validation, resources, formal analysis, writing‐reviewing and editing. **Marco Fiorillo:** Validation, formal analysis, resources, data curation, writing‐reviewing and editing. **Alessia Gianferrari:** Validation, investigation, formal analysis, writing‐reviewing and editing. **Noemi Puccio:** Writing‐reviewing and editing. **Antonino Neri:** Resources, writing‐reviewing, and editing. **Alessia Ciarrocchi:** Resources, writing‐reviewing and editing, funding acquisition. **Mariaelena Pistoni:** Conceptualization, methodology, validation, formal analysis, investigation, writing‐original draft, writing‐reviewing and editing, supervision, project administration.

## FUNDING INFORMATION

A.C. is supported by Fondazione AIRC per la Ricerca sul Cancro Grant number AIRC IG21772. N.A. is supported by Fondazione AIRC per la Ricerca sul Cancro Grant number IG24449. This study was partially supported by the Italian Ministry of Health—Ricerca Corrente Annual Program 2025. M.E.G.C. is supported by the PNRR M4C2‐Investimento 1.4‐CN00000041 finanziato dall'Unione europea—NextGenerationEU.

## CONFLICT OF INTEREST STATEMENT

The authors declare that they have no known competing financial interests or personal relationships that could have appeared to influence the work reported in this paper.

## Supporting information


**Supplementary Figure 1.** (A) Schematic representation of the cell death pathways deregulated after BETi treatment. Red arrows mean downregulation. (B), (C) Quantification of western blots bands of cMyc, BCL2 and CytC protein expression in TNBC cell lines treated with JQ1 (B) or OTX (C). (D) ROS measurement in MB231, Hs578t and BT549 cells after 24 h of BETi administration alone or in combination with N‐acetylcysteine (NAC). Results were compared to control (CNT). CNT+ stands for positive control, ns stands for not significant. (E) Western blot images of TNBC cells treated with JQ1 and OTX for 24 h. Blots are probed with antibodies against SOD1 and β‐actin (bACT). (F)–(H) Quantification of western blot bands of MFF, pDRP1, and DRP1 protein expression in TNBC cell lines treated with BETi and analysis of the ratio between pDRP1 and DRP1 expression in MB231 (F), BT549 (G) and Hs578t (H). (I) Cell proliferation assay measured by IncuCyte analysis to observe the effect of different concentrations (5–10‐20 μM) of Mdivi‐1 on BT549 cells over 108 hours of treatment. (J) On the left, a schematic timeline of the experiment and treatment. On the right, western blot images of MB231 and Hs578t cells transfected with EV or DRP1 treated with BETi compared to control (CNT). Blots are probed with antibodies against DRP1 and β‐actin (bACT). (K) Percentage of apoptotic cells in MB231 and Hs578t cells transfected with DRP1 and treated with BETi (JQ1, J and OTX, O) with Annexin V staining. The significance is calculated versus the EV/CNT treated cell (*, **) and DRP1/CNT treated cells (#, ##). (L) The mtDNA content quantification of MCF7 cells treated with JQ1 and OTX over 2 days of treatment. The results are presented as a fold change of treated cells to the relative control (CNT). (M) Western blot images of TNBC cells treated with JQ1 (above) and OTX (below) over time for 2 days. Blots are probed with antibodies against Tom20 and β‐actin (bACT). The original SDS‐PAGE membrane of β‐actin is the same as in Figure 2A, B. BETi were used at a final concentration of 1 μM.
Supplementary Figure 2. (A), (B) Table of amino acid levels represented as a mean ±SD. In yellow the amino acids level statistically significant between treatment and control in MB231 (A) and Hs578t (B). (C) Western blot images of TNBC cells treated with JQ1 and OTX over 24 hs compared to control (CNT). Blots are probed with antibodies against ACC and β‐actin (bACT). (D) Western blot images of BT549 cells treated with JQ1 and OTX for 24 h. Blots are probed with antibodies against SDHa, PDHa, ACC, and β‐actin (bACT). (E) Western blot images of MB231 and Hs578t cells after transfection of control (siCNT) or siBRD4 (siB4) at day 3 (3 d) and day 4 (4 d). Blots are probed with antibodies against BRD4, BCL2, and β‐actin (bACT). The original SDS‐PAGE membranes of BRD4 and β‐actin are the same as in Figure 4B. (F) Quantification of western blot bands of MFF, pDRP1, and DRP1 protein expression in TNBC cell lines interfered with siRNA BRD4 and analysis of the ratio between pDRP1 and DRP1 expression. (G) Western blot images of BT549 cells treated with 5 and 10 μM of Mdivi‐1 for 24 h compared to control (CNT). Blots are probed with antibodies against SDHa and β‐actin (bACT). BETi were used at a final concentration of 1 μM.

## Data Availability

Data sharing not applicable to this article as no datasets were generated or analyzed during the current study.
