# Supplementary material for: BET inhibitors (BETi) influence oxidative phosphorylation metabolism by affecting mitochondrial dynamics leading to alterations in apoptotic pathways in triple‐negative breast cancer (TNBC) cells
Source: Cell Prolif. 2024 Sep 2;57(12):e13730. doi: 10.1111/cpr.13730 (PMC11628750; doi:10.1111/cpr.13730)
Supplement: Supplementary file 1 — Supplementary Figure 1. (A) Schematic representation of the cell death pathways deregulated after BETi treatment. Red arrows mean downregulation. (B), (C) Quantification of western blots bands of cMyc, BCL2 and CytC protein expression in TNBC cell lines treated with JQ1 (B) or OTX (C). (D) ROS measurement in MB231, Hs578t and BT549 cells after 24 h of BETi administration alone or in combination with N‐acetylcysteine (NAC). Results were compared to control (CNT). CNT+ stands for positive control, ns stands for not significant. (E) Western blot images of TNBC cells treated with JQ1 and OTX for 24 h. Blots are probed with antibodies against SOD1 and β‐actin (bACT). (F)–(H) Quantification of western blot bands of MFF, pDRP1, and DRP1 protein expression in TNBC cell lines treated with BETi and analysis of the ratio between pDRP1 and DRP1 expression in MB231 (F), BT549 (G) and Hs578t (H). (I) Cell proliferation assay measured by IncuCyte analysis to observe the effect of different concentrations (5–10‐20 μM) of Mdivi‐1 on BT549 cells over 108 hours of treatment. (J) On the left, a schematic timeline of the experiment and treatment. On the right, western blot images of MB231 and Hs578t cells transfected with EV or DRP1 treated with BETi compared to control (CNT). Blots are probed with antibodies against DRP1 and β‐actin (bACT). (K) Percentage of apoptotic cells in MB231 and Hs578t cells transfected with DRP1 and treated with BETi (JQ1, J and OTX, O) with Annexin V staining. The significance is calculated versus the EV/CNT treated cell (*, **) and DRP1/CNT treated cells (#, ##). (L) The mtDNA content quantification of MCF7 cells treated with JQ1 and OTX over 2 days of treatment. The results are presented as a fold change of treated cells to the relative control (CNT). (M) Western blot images of TNBC cells treated with JQ1 (above) and OTX (below) over time for 2 days. Blots are probed with antibodies against Tom20 and β‐actin (bACT). The original SDS‐PAGE membrane of β‐actin [file CPR-57-e13730-s001.pptx]

## Slide 1
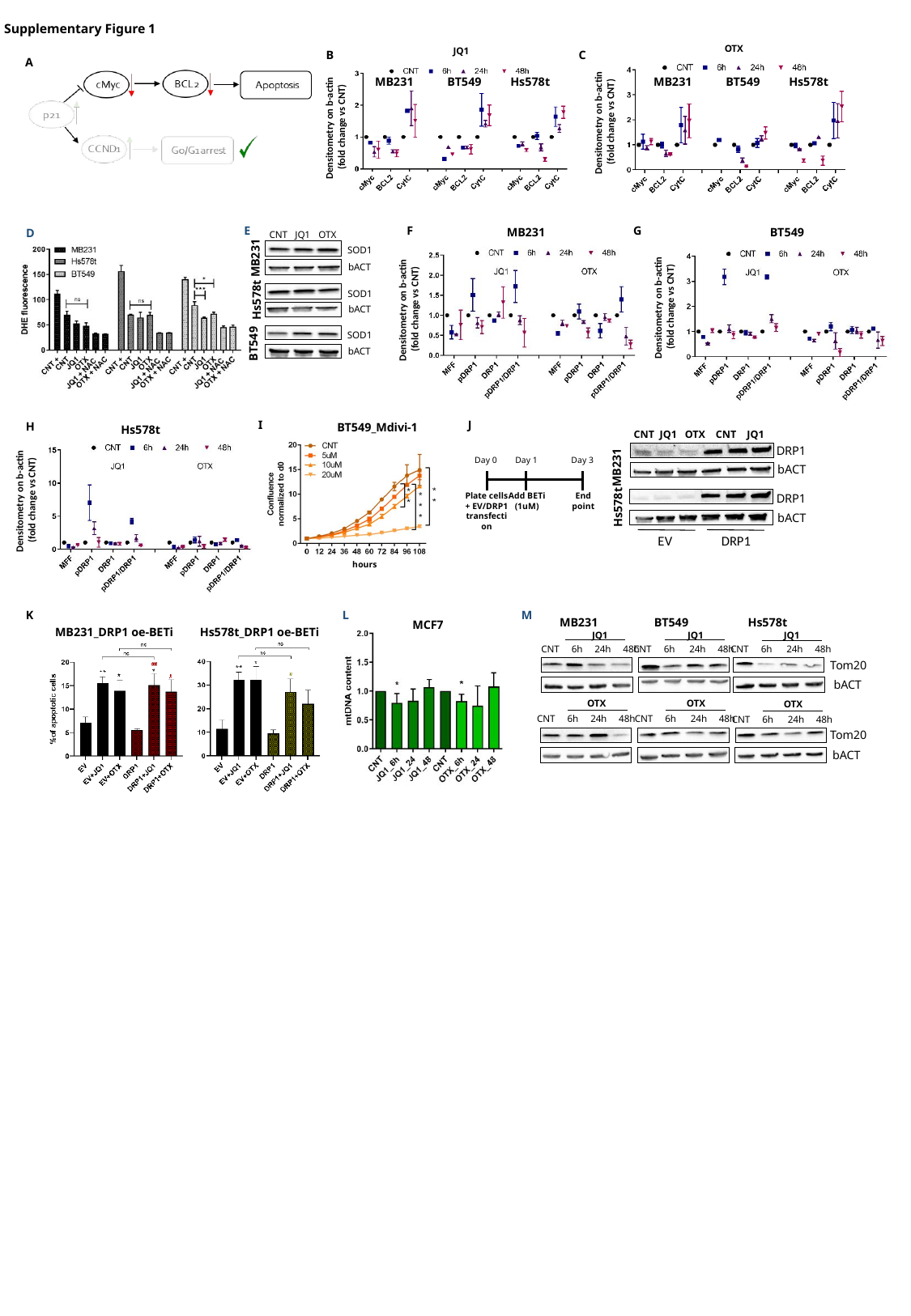

Supplementary Figure 1
OTX
JQ1
B
C
A
MB231
BT549
Hs578t
MB231
BT549
Hs578t
Densitometry on b-actin
 (fold change vs CNT)
Densitometry on b-actin
 (fold change vs CNT)
E
F
G
MB231
BT549
D
CNT
JQ1
OTX
SOD1
MB231
bACT
SOD1
Hs578t
Densitometry on b-actin
 (fold change vs CNT)
Densitometry on b-actin
 (fold change vs CNT)
bACT
SOD1
BT549
bACT
I
J
H
BT549_Mdivi-1
Hs578t
CNT JQ1 OTX CNT JQ1 OTX
DRP1
Day 0
Day 1
Day 3
Plate cells + EV/DRP1 transfection
Add BETi
(1uM)
End point
MB231
bACT
Densitometry on b-actin
 (fold change vs CNT)
DRP1
Hs578t
bACT
EV
DRP1
hours
K
L
M
MB231
BT549
Hs578t
MCF7
MB231_DRP1 oe-BETi
Hs578t_DRP1 oe-BETi
JQ1
JQ1
JQ1
CNT 6h 24h 48h
CNT 6h 24h 48h
CNT 6h 24h 48h
Tom20
bACT
OTX
OTX
OTX
CNT 6h 24h 48h
CNT 6h 24h 48h
CNT 6h 24h 48h
Tom20
bACT

## Slide 2
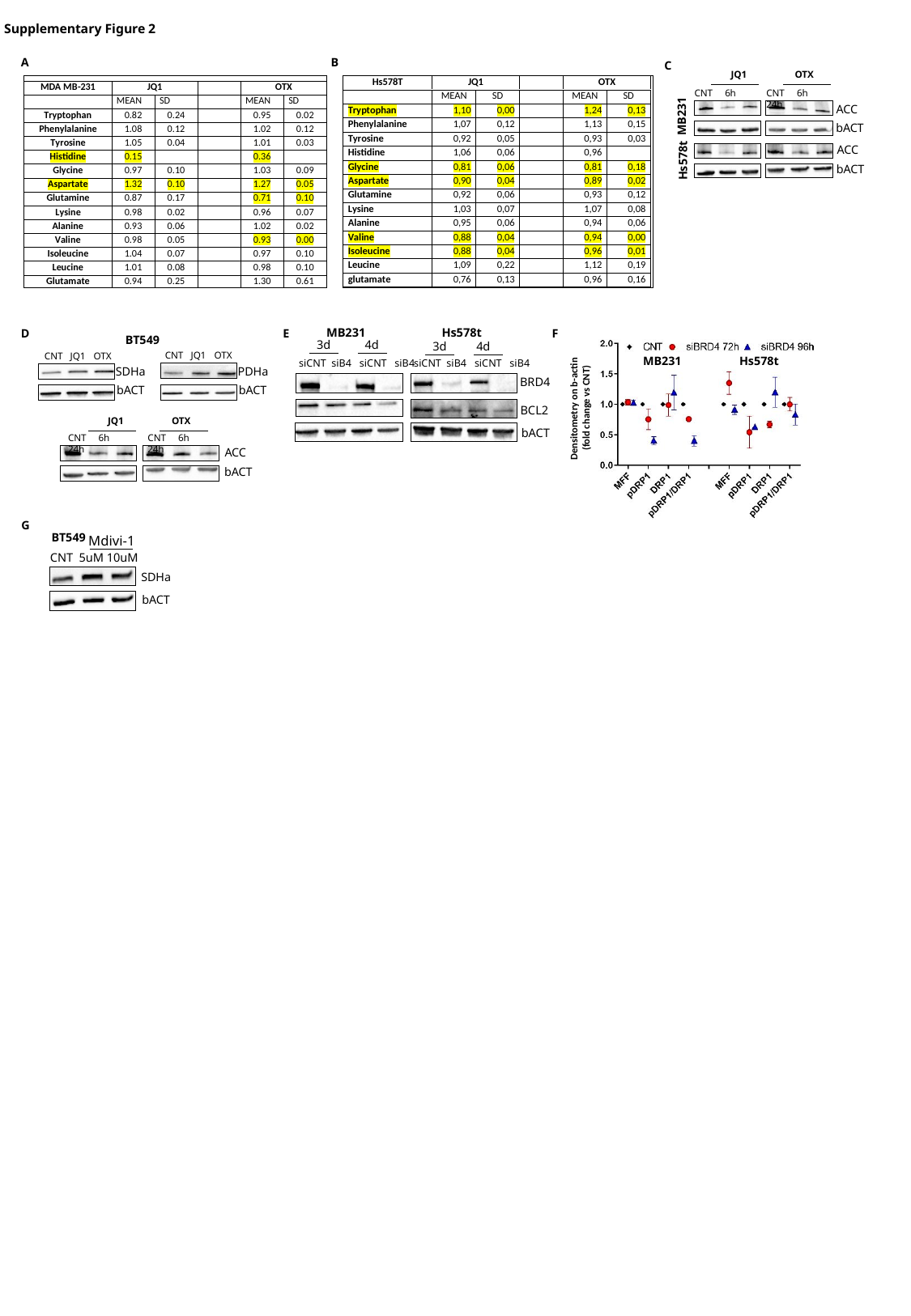

Supplementary Figure 2
A
B
C
JQ1
OTX
CNT 6h 24h
CNT 6h 24h
ACC
MB231
bACT
ACC
Hs578t
bACT
MB231
Hs578t
D
E
F
BT549
3d
4d
3d
4d
CNT
JQ1
OTX
CNT
JQ1
OTX
MB231
Hs578t
siCNT siB4 siCNT siB4
siCNT siB4 siCNT siB4
SDHa
PDHa
BRD4
bACT
bACT
Densitometry on b-actin
 (fold change vs CNT)
BCL2
JQ1
OTX
bACT
CNT 6h 24h
CNT 6h 24h
ACC
bACT
G
BT549
Mdivi-1
CNT 5uM 10uM
SDHa
bACT
